# Educational Mobility, Pace of Aging, and Lifespan Among Participants in the Framingham Heart Study

**DOI:** 10.1001/jamanetworkopen.2024.0655

**Published:** 2024-03-01

**Authors:** Gloria H. J. Graf, Allison E. Aiello, Avshalom Caspi, Meeraj Kothari, Hexuan Liu, Terrie E. Moffitt, Peter A. Muennig, Calen P. Ryan, Karen Sugden, Daniel W. Belsky

**Affiliations:** 1 Department of Epidemiology, Columbia University Mailman School of Public Health, New York, New York; 2 Robert N. Butler Columbia Aging Center, New York, New York; 3 Department of Psychology & Neuroscience, Duke University, Durham, North Carolina; 4 Department of Psychiatry and Behavioral Sciences, Duke University School of Medicine, Durham, North Carolina; 5 PROMENTA, University of Oslo, Oslo, Norway; 6 Social, Genetic & Developmental Psychiatry Centre, Institute of Psychiatry, Psychology, & Neuroscience, King’s College London, London, United Kingdom; 7 School of Criminal Justice, University of Cincinnati, Cincinnati, Ohio; 8 Institute for Interdisciplinary Data Science, University of Cincinnati, Cincinnati, Ohio; 9 Department of Health Policy and Management, Columbia University Mailman School of Public Health, New York, New York

## Abstract

**Importance:**

People who complete more education live longer lives with better health. New evidence suggests that these benefits operate through a slowed pace of biological aging. If so, measurements of the pace of biological aging could offer intermediate end points for studies of how interventions to promote education will affect healthy longevity.

**Objective:**

To test the hypothesis that upward educational mobility is associated with a slower pace of biological aging and increased longevity.

**Design, Setting, and Participants:**

This prospective cohort study analyzed data from 3 generations of participants in the Framingham Heart Study: (1) the original cohort, enrolled beginning in 1948; (2) the Offspring cohort, enrolled beginning in 1971; and (3) the Gen3 cohort, enrolled beginning in 2002. A 3-generation database was constructed to quantify intergenerational educational mobility. Mobility data were linked with blood DNA-methylation data collected from the Offspring cohort in 2005 to 2008 (n = 1652) and the Gen3 cohort in 2009 to 2011 (n = 1449). Follow-up is ongoing. Data analysis was conducted from June 2022 to November 2023 using data obtained from the National Institutes of Health database of Genotypes and Phenotypes (dbGaP).

**Exposure:**

Educational mobility was measured by comparing participants’ educational outcomes with those of their parents.

**Main Outcomes and Measures:**

The pace of biological aging was measured from whole-blood DNA-methylation data using the DunedinPACE epigenetic clock. For comparison purposes, the analysis was repeated using 4 other epigenetic clocks. Survival follow-up was conducted through 2019.

**Results:**

This study analyzed data from 3101 participants from the Framingham Heart Study; 1652 were in the Offspring cohort (mean [SD] age, 65.57 [9.22] years; 764 [46.2%] male) and 1449 were in the Gen3 cohort (mean [SD] age, 45.38 [7.83] years; 691 [47.7%] male). Participants who were upwardly mobile in educational terms tended to have slower pace of aging in later life (*r* = −0.18 [95% CI, −0.23 to −0.13]; *P* < .001). This pattern of association was similar across generations and held in within-family sibling comparisons. There were 402 Offspring cohort participants who died over the follow-up period. Upward educational mobility was associated with lower mortality risk (hazard ratio, 0.89 [95% CI, 0.81 to 0.98]; *P* = .01). Slower pace of aging accounted for approximately half of this association.

**Conclusions and Relevance:**

This cohort study’s findings support the hypothesis that interventions to promote educational attainment may slow the pace of biological aging and promote longevity. Epigenetic clocks have potential as near-term outcome measures of intervention effects on healthy aging. Experimental evidence is needed to confirm findings.

## Key Points

**Question:**

Is upward educational mobility associated with a slower pace of biological aging and increased longevity?

**Findings:**

In this cohort study of 3101 participants representing 2 generations of the Framingham Heart Study, upward educational mobility was associated with a slower pace of aging (as measured with whole-blood DNA-methylation data) and lower risk of death. Slower pace of aging accounted for approximately half of the association between educational mobility and mortality.

**Meaning:**

These results suggest that interventions to promote educational attainment may slow the pace of biological aging and promote longevity.

## Teaser

This cohort study examines whether upward educational mobility is associated with a slower pace of biological aging and increased longevity among 2 generations of participants in the Framingham Heart Study (FHS).

## Introduction

People who complete more years of schooling tend to live longer, healthier lives. This educational gradient is thought to arise through improvements in socioeconomic resources and resulting access to health services, health-promoting social networks and communities, and healthy behaviors.^1,2^ Educational gradients are apparent in nearly every organ system and aging-related disease, including heart disease, diabetes, cancer, Alzheimer disease, and so on^3,4,5,6,7,8,9,10,11^; people with higher levels of education experience a lower prevalence of aging-related disease and later age of onset of disease. This evidence of more rapid decline across organ systems suggests an overall acceleration of the pace of biological aging.

Biological aging refers to a set of processes characterized by an accumulation of molecular changes or hallmarks that progressively undermine the integrity and resilience capacity of our cells, tissues, and organs as we grow older.^12,13^ We recently developed a novel method to quantify the pace of biological aging in humans. Our approach used longitudinal phenotyping of multiorgan system decline to derive a DNA-methylation (DNAm) blood test measurement of the pace of biological aging, DunedinPACE (pace of aging calculated in the epigenome).^14^

We recently found that the pace of aging, as measured by the DunedinPACE epigenetic clock, was accelerated in individuals with low levels of education, and slowed in those with higher levels of education.^15,16^ In this study, we build on these observations to test the hypothesis that higher educational attainment promotes longevity by slowing the pace of aging. Because genetic and social inheritances affect how much education a person completes^17^ and may also affect their pace of aging,^18^ we focused analysis on educational mobility (ie, differences in education of children relative to their parents). We further conducted analysis of sibling differences to address potential confounding by other factors shared within families.^19^ These designs help isolate associations of education with the pace of aging from effects of correlated family-level factors.

We measured participants’ educational mobility by linking records across 3 generations of Framingham Heart Study (FHS) participants. This procedure allowed us to compute educational mobility for members of the 2 most recent generations (Offspring and Gen3 cohorts). For these participants, we measured pace of aging from blood DNAm using the DunedinPACE epigenetic clock. For the Offspring cohort, we also measured survival over 15 years of follow-up. Analysis proceeded in 2 steps. We first tested associations of educational mobility with pace of aging and survival. We then tested mediation of mobility-mortality associations through pace of aging. This analytic framework allowed us to test the hypothesis that slower pace of aging mediates the association of upward educational mobility with increased longevity.

## Methods

Study protocols and results were reported following the Strengthening the Reporting of Observational Studies in Epidemiology (STROBE) reporting guidelines for cohort studies. Analysis of Database of Genotypes and Phenotypes (dbGaP) data was approved by the Columbia University Medical Center institutional review board. Informed consent was waived because deidentified FHS data were accessed through the dbGaP.

### Data and Participants

The FHS is an ongoing observational cohort study first initiated in 1948, spanning 3 generations. The original cohort was a two-thirds population sample of Framingham, Massachusetts, and aimed to identify risk factors for cardiovascular disease. Additional cohorts have since been recruited, consisting of the children and grandchildren of the original cohort and their spouses. DNAm data were available from blood tests administered during Offspring cohort examination 8 (2005-2008) and Gen3 cohort examination 2 (2009-2011).

**Figure 1.**
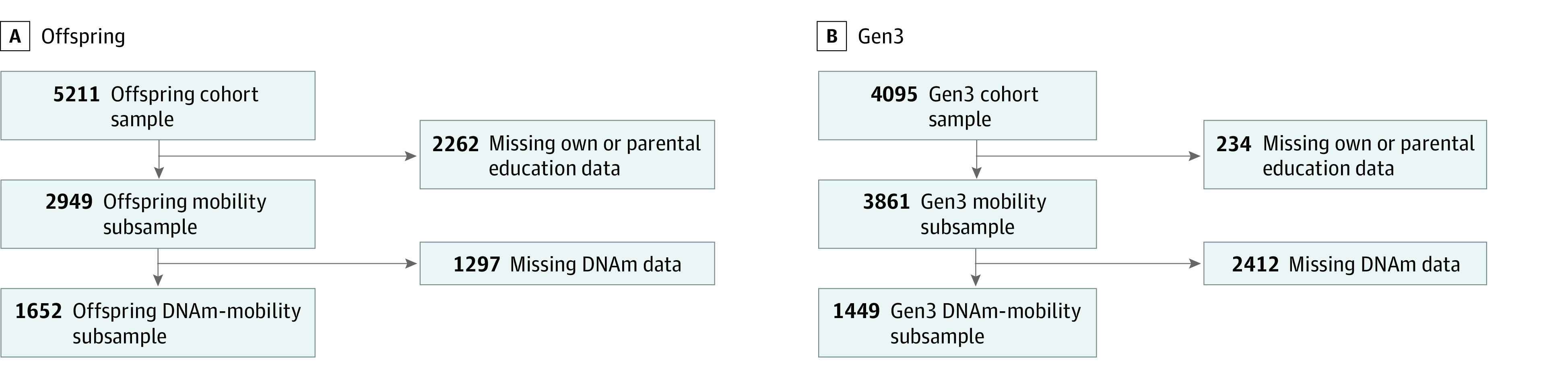
Offspring and Gen3 Participant Flow Diagrams The figure shows how the final analytic samples were developed from the larger set of all participants in the Offspring (n = 1652) and Gen3 (n = 1449) Framingham Heart Study cohorts (combined n = 3101). DNAm indicates DNA-methylation blood test.

We analyzed data from all generations of the FHS (n = 14 106 with available education data). Our DNAm analysis sample consisted of participants with available education data who could be linked to educational data from at least 1 parent and who provided a blood sample for DNAm analysis. This sample included 1652 members of the Offspring cohort from 1025 families and 1449 members of the Gen3 cohort from 552 families (Figure 1). Race and ethnicity were not assessed because more than 99% of the patients in the FHS were White.

### Educational Mobility

#### Educational Attainment

Participants reported their highest level of education to interviewers. For analysis, we converted levels to years of schooling and standardized values within sex and 5-year birth cohort to account for secular trends in educational attainment (details in the eMethods in Supplement 1).

#### Educational Mobility

Educational attainments were correlated between parents and their children (Pearson *r* = 0.35; *P* < .001) (eFigure 1 in Supplement 1). We computed mobility values using residualized-change scores, which quantify mobility as the difference between a participant’s educational attainment and the attainment expected based on the educational levels of their parents, and difference scores, which quantify mobility as the raw difference between parental and Offspring educational attainment.^16^ Both metrics are denominated in sex-standardized and birth cohort–standardized units of education which, on average, corresponded to approximately 2 years of schooling.

### Biological Aging

Whole-genome DNAm profiles were obtained from dbGaP (phs000724.v9.p13). Details are reported in the eMethods in Supplement 1.

#### DunedinPACE

Biological aging is the progressive loss of integrity and resilience capacity in our cells, tissues, and organs that occurs with advancing chronological age.^20,21^ Pace of aging is a phenotype reflecting the rate at which these biological changes occur.^22^ We quantified pace of aging from DNAm using the DunedinPACE epigenetic clock algorithm.^14^ This algorithm was developed from analysis of a 20-year longitudinal change in 19 biomarkers of organ-system integrity in the Dunedin Study 1972-to-1973 birth cohort at ages 26 years, 32 years, 38 years, and 45 years. A longitudinal pace-of-aging phenotype derived from this analysis^23^ was then distilled into a single-time point DNAm blood test using elastic-net regression.^24^ In diverse cohorts in the US and around the globe, this algorithm has been associated with incident morbidity and disability, survival, and a range of socioenvironmental exposures including educational attainment.^14,15,25,26,27,28,29,30,31,32^ We calculated participants’ clock values from their DNAm data calculated using code available on GitHub.^33^

#### Other Epigenetic Clocks

Other candidate measures of aging can be computed from DNAm data. For comparison purposes, we repeated analysis using 4 alternative epigenetic clocks widely studied in the literature and included in our prior articles: the Horvath, Hannum, PhenoAge, and GrimAge clocks.^34,35,36,37^ These clocks were calculated using the online calculator hosted by the Horvath Lab.^38^ Clock values were residualized for chronological age prior to analysis.

### Survival

Details of FHS survival and mortality follow-up are reported elsewhere.^39^ Briefly, FHS conducts continuous mortality follow-up for all study participants. Date and cause of death are recorded for each participant based on hospital records, death certificates, and next-of-kin interviews. The present study included mortality data accumulated through 2019 (mean follow-up from DNAm baseline was approximately 12 years).

### Statistical Analysis

We tested associations of educational mobility with pace of biological aging as measured by DunedinPACE using linear regression models. We used generalized estimating equations to account for nonindependence of observations of individuals within nuclear families.^40^ We conducted within-family analysis comparing sibling differences in educational attainment with sibling differences in pace of aging using fixed effects regression.^41^ We tested associations of educational mobility and pace of aging with survival time using Cox proportional hazard regression models. Mediation analysis was conducted using the CMAverse package^42^ in R version 4.0.3 (R Project for Statistical Computing)^43^ following the approach described by Valeri and Vanderweele.^44^ Clock values were standardized to a mean (SD) of 0 (1) for analysis. For regression models, significance testing for model coefficients was conducted using 2-sided *t* tests at the *P* < .05 level. For mediation analysis, we used a regression-based estimation approach with bootstrap standard errors to obtain 95% CIs. All models were adjusted for age and sex. Statistical analysis was performed from June 2022 to November 2023.

## Results

We analyzed data from 3101 participants from the Offspring cohort (n = 1652; mean [SD] age at DNAm measurement, 65.57 [9.22] years; 764 [46.2%] male) and Gen3 cohort (n = 1449; mean [SD] age at DNAm measurement, 45.38 [7.83] years; 691 [47.7%] male) of the Framingham Heart Study (FHS). Offspring cohort participants completed a mean (SD) of 14.74 (2.31) years of education, which was approximately 2 years more than their highest-educated parent (mean [SD] years of education, 12.35 [2.45] years). Gen3 cohort participants completed a mean (SD) of 15.24 (1.88) years of education, which was similar with their highest-educated parent (mean [SD] years of education, 14.98 [2.26] years). In the Offspring cohort, 402 (24.3%) died over the 15-year follow-up period. Participant characteristics are reported in the Table. Participants with data on education and educational mobility were similar with the overall DNAm sample.

**Table.** Characteristics of DNA-Methylation, Education, and Mobility Samples^a^

Characteristic	Analytic sample (n = 3101)	Offspring sample (n = 1652)	Gen3 sample (n = 1449)
Families, No.	1577	1025	552
Age, mean (SD), y	56.14 (13.25)	65.57 (9.22)	45.38 (7.83)
Sex, No. (%)			
Female	1646 (53.1)	888 (53.8)	758 (52.3)
Male	1455 (46.9)	764 (46.2)	691 (47.7)
Died, No. (%)	419 (13.5)	402 (24.3)	NA
Education, mean (SD), y	14.97 (2.13)	14.74 (2.31)	15.24 (1.88)
Parental education, mean (SD), y	13.58 (2.70)	12.35 (2.45)	14.98 (2.26)
Educational mobility (Δ), y	1.27 (2.66)	2.22 (2.62)	0.18 (2.27)
Educational mobility (RC), y	0.24 (1.98)	0.43 (2.15)	0.02 (1.73)
Educational mobility (Δ, standardized)	−0.19 (1.13)	−0.03 (1.17)	−0.38 (1.06)
Educational mobility (RC, standardized)	0.08 (0.91)	0.17 (0.90)	−0.02 (0.91)
DunedinPACE	1.06 (0.12)	1.08 (0.12)	1.03 (0.11)

Abbreviations: NA, not applicable; RC, residualized change.

^a^ The table provides information on the composition of our analytic sample (n = 3101, Offspring n = 1652, Gen3 n = 1449). The analytic sample includes all individuals who provided DNA-methylation data, who reported their own educational attainment levels, and for whom educational attainment data was available for at least 1 parent.

### Educational Mobility and Pace of Biological Aging

**Figure 2.**
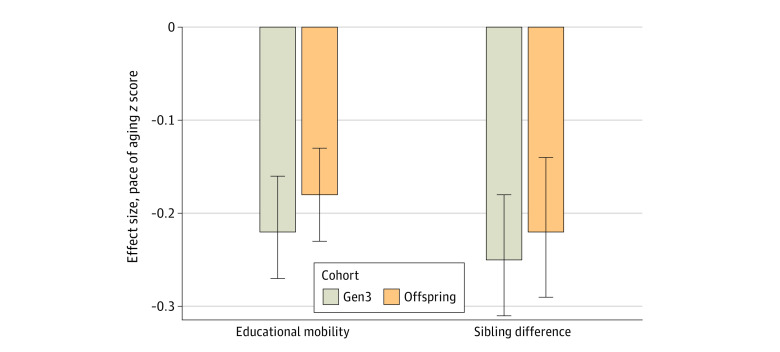
Association of Educational Mobility With Pace of Aging The figure shows effect estimates for associations of educational mobility with pace of aging as measured with the DunedinPACE epigenetic clock. The bars on the left show differences in pace of aging *z* score per 1-SD unit of upward educational mobility (Offspring effect size [ES], −0.17; *P* < .001; Gen3 ES, −0.21; *P* < .001). The bars on the right show differences in pace of aging *z* score per 1-SD difference in educational attainment between siblings, as estimated using family fixed-effects regression (Offspring ES, −0.21; *P* < .001; Gen3 ES, −0.25; *P* < .001). All models included covariate adjustment for participant age and sex.

Participants who were upwardly mobile had slower pace of aging than those who were downwardly mobile (for residualized-change mobility, Offspring cohort Cohen *d* = −0.17 [95% CI, −0.22 to −0.12]; *P* < .001; Gen3 cohort Cohen *d* = −0.21 [95% CI, −0.26 to −0.15]; *P* < .001; for difference-score mobility, Offspring cohort Cohen *d* = −0.06 [95% CI, −0.10 to −0.02]; *P* = .002; Gen3 cohort Cohen *d* = −0.07 [95% CI, −0.11 to −0.02]; *P* = .006) (Figure 2; eTable 1, eFigure 3 in Supplement 1). As a sensitivity analysis, we tested consistency of associations across participants who were born into lower- and higher-educated families to evaluate whether returns to educational mobility were concentrated at one end of the socioeconomic continuum. Effect sizes were similar across strata of parental education (eFigure 2 in Supplement 1). In addition, effect sizes for educational mobility were comparable across Offspring and Gen3 cohorts, suggesting consistent returns to relative educational mobility over time (Figure 2; eFigure 3 in Supplement 1). In comparative analysis of other epigenetic clocks, associations were weaker and not statistically different from 0 for the Horvath, Hannum, and PhenoAge clocks. Results for the GrimAge epigenetic clock, which was developed within the FHS, were similar to those for DunedinPACE. Full results are reported in eTable 1 in Supplement 1.

### Sibling Differences in Educational Attainment and Pace of Biological Aging

To the extent that there are social or environmental factors that affect both educational mobility and aging trajectories, our results may overstate the association of mobility and healthy aging. To address this potential confounder, we repeated our analysis within families. For participants with a sibling in the data (n = 2437; Offspring cohort n = 1096; Gen3 cohort n = 1341), we tested whether the difference in educational mobility between siblings was associated with differences in the pace of biological aging. This design blocks confounding by all factors shared by siblings in a family. Results were similar to our primary analysis. The sibling with higher educational mobility tended to have slower pace of aging as measured by the DunedinPACE epigenetic clock as compared with their less-educated sibling (Offspring cohort Cohen *d* = −0.21 [95% CI, −0.28 to −0.13]; *P* < .001; Gen3 cohort Cohen *d* = −0.25 [95% CI, −0.32 to −0.19]; *P* < .001) (eTable 2 in Supplement 2). Again, results were not statistically different from 0 for the Horvath, Hannum, and PhenoAge epigenetic clocks. Results for the GrimAge epigenetic clock, which was developed within the FHS, were similar to those for the DunedinPACE epigenetic clock (eTable 2 in Supplement 2).

### Educational Mobility, Pace of Aging, and Longevity

We next focused our attention on educational gradients in mortality in the Offspring cohort (n = 1652; Gen3 participants were not included in this analysis because very few deaths occurred in this younger cohort during the follow-up period). Participants who were more upwardly educationally mobile had lower mortality risk (for residualized-change mobility: hazard ratio [HR], 0.87 [95% CI, 0.79 to 0.96]; *P* = .047; for difference-score mobility: HR, 0.92 [95% CI, 0.83 to 1.02]; *P* = .10). In parallel, as previously reported,^14^ participants with faster pace of aging were at higher risk of death than those with a slower pace of aging (mortality HR, 1.61 [95% CI, 1.49 to 1.74]; *P* < .001). All DNAm clocks, with the exception of the Horvath clock, were associated with mortality; effect sizes were attenuated relative to DunedinPACE with the exception of the GrimAge clock, which was developed to estimate mortality in the FHS sample. Full results are reported in eTable 3 in Supplement 1.

### Mediation Analysis of Educational Gradients in Mortality by Pace of Aging

Finally, we tested whether differences in pace of aging mediated educational gradients in mortality risk. We found that DunedinPACE mediated 50% of the association between educational mobility and mortality risk (indirect effect HR, 0.93 [95% CI, 0.90 to 0.95]). Results were robust to methods that allow relaxation of assumptions about exposure-mediator and mediator-outcome confounding and exposure-mediator interactions.^44^ Full results are reported in eTable 4 in Supplement 2.

### Sensitivity Analyses

Pace of aging was measured from blood DNAm data. Blood DNAm is affected by smoking history and DNA-sample white-blood-cell composition.^45,46^ In turn, these factors may relate to mortality risk. Therefore, we repeated analysis including covariate adjustment for these factors. Smoking history was recorded from participant reports; white blood cell composition in the DNA sample was estimated using the algorithms proposed by Houseman and colleagues.^47^ Covariate adjustment for estimated cell counts and participant reports of smoking history resulted in modest attenuation of some effect sizes; however, all analyses showed substantial mediation of educational gradients in mortality risk by pace of aging, measured using DunedinPACE (eTable 4 in Supplement 2). Full results are reported in eTables 5, 6, 7, and 8 in Supplement 1. Finally, we repeated our core analysis using unstandardized versions of the education and mobility variables. Results were similar to those reported in the main text (eTable 9 in Supplement 1).

## Discussion

People with higher levels of education tend to live longer, healthier lives as compared with those with less education.^48,49,50^ We analyzed data from 3 generations of the FHS to test whether this educational gradient in healthspan and lifespan could reflect effects of education on the pace of biological aging. Participants who were upwardly mobile in educational terms had slower pace of aging, as measured by the DunedinPACE epigenetic clock and were less likely to die over the follow-up period. Differences in pace of aging accounted for roughly half of the association between educational mobility and mortality risk.

DunedinPACE was developed as a surrogate end point for interventions targeting healthy lifespan.^51,52,53^ Prior studies have reported associations between education and DunedinPACE, and between DunedinPACE and aging-related disease and mortality.^14,15,25,27^ Our study, to our knowledge, is the first to follow individuals across the educational-origins to educational-attainment to pace-of-aging to mortality pathway. The magnitude of associations between education and pace of biological aging we report in this sample (*r* of 0.19 to 0.24) are consistent with population-representative studies in the US, UK, and New Zealand (*r* of 0.17 to 0.38^15^) and correspond to a 2% to 3% slower pace of aging per unit of upward educational mobility (equivalent to approximately 2 years of additional schooling). In turn, our mediation analysis found that this magnitude of slowing in pace of aging corresponded to an approximately 7% reduction in the hazard of mortality, half of the overall effect of educational mobility. Collectively, these findings contribute evidence that DunedinPACE is a candidate surrogate end point for the association of educational interventions with aging.

A further contribution of our study is evidence that healthy-aging returns to education persist into more recent birth cohorts, among whom higher levels of education are more common. Educational gradients in mortality have grown steeper in recent years.^54^ However, these trends reflect outcomes primarily for the cohorts born across the early-to-middle 20th century. Across these cohorts, the proportion of individuals completing high school and college education increased dramatically.^55^ Whether the trend of widening educational inequality in healthy aging will persist for later-20th century birth cohorts, for whom rates of high school and college graduation have been more stable, is unknown. We found that effect sizes for associations between upward educational mobility and slower pace of aging were similar for the Offspring and Gen3 cohorts, suggesting that even in the context of relatively high educational attainment, upward mobility continues to yield returns for healthy aging.

### Limitations

We acknowledge that this study has limitations. There is no criterion standard measure of biological aging.^21^ We focused on the pace of aging measure DunedinPACE based on 3 lines of evidence. First, the DunedinPACE algorithm is predictive of diverse aging-related outcomes, including disease, disability, and mortality.^14,25,26,27,29^ Second, the algorithm is associated with social determinants of healthy aging in young, midlife, and older adults.^14,15,28,30,31,56^ Third, the algorithm shows evidence of being modified by calorie restriction,^57^ an intervention that modifies the basic biology of aging in animal experiments.^58^ Confidence in results is further supported by the consistency of our findings with those for alternative measurements of biological aging in independent cohorts.^59^ Additionally, our results are robust to known confounds of DNAm-based measurements of aging, specifically cell composition of blood samples used to derive DNA and smoking history.^46,60^

There are many factors that may drive both educational attainment and slower biological aging, such as childhood poverty.^18,28,56,61,62^ Confounding by such factors would lead simple associations to overstate the potential of education interventions to modify biological aging. We addressed this threat of confounding using 2 designs that control for differences between participants in their family history and early-life environment. First, we analyzed educational mobility between generations of a family. Second, we analyzed differences between siblings within a family. Across these specifications, we found consistent evidence of slower pace of aging in people who were upwardly educationally mobile and who completed more schooling as compared with their siblings. Ultimately, evidence from randomized trials^63^ is needed to confirm whether promoting educational attainment slows the pace of aging. Furthermore, the path from educational mobility to healthy longevity involves posteducation attainments. Studies of mediating mechanisms, including income and wealth accumulation, occupational characteristics, health literacy, and health care access, can help refine understanding of how upward educational mobility slows the pace of aging.^64,65^

The FHS is predominantly White-identifying and includes relatively few participants who did not graduate from high school. In data sets representative of the United States population, the difference in mortality risk associated with having attended some college as compared with not is HR of 0.66 to 0.76.^66^ In the data we analyzed, the corresponding effect size is smaller (HR, 0.81). Underrepresentation of individuals with low educational attainment could attenuate FHS education-mortality associations. This bias should make our estimates of mediation by DunedinPACE conservative. Nevertheless, replication in more diverse cohorts is a priority. The FHS Offspring cohort DNAm measurement occurred at the 8th examination, after approximately 4 decades of follow-up. Survival bias could affect results. However, we observed similar effect sizes for associations of educational mobility with pace of aging in the younger Gen3 cohort, for whom DNAm data were generated from samples collected at their 2nd examination.

## Conclusions

The healthier aging of individuals with more education and other social advantages is well established. In this prospective cohort study of educational mobility in 2 generations of the FHS, we found that an accelerated pace of biological aging is associated with this inequality. In addition, findings suggest that new methods to quantify the pace of aging can provide near-term measures of health effects for programs and policies designed to promote educational attainment and other socioeconomic assets. Because the pace of aging is variable from young adulthood, measurements such as DunedinPACE can potentially illuminate intervention effects years or decades before aging-related functional deficits and chronic diseases become apparent. Such information can, in turn, help refine efforts to heal health disparities and build aging health equity.

## Notes

Supplement 1. **eMethods.** Supplemental Methods **eTable 1.** Associations of Educational Attainment, Parental Education, and Educational Mobility With Biological Aging in Offspring and Gen3 Framingham Study Participants **eTable 2.** Fixed-Effects Regression of Biological Aging on Education Within Sibling Clusters **eTable 3.** Associations of Biological Aging, Educational Attainment, and Educational Mobility With Overall Survival in the Framingham Offspring Cohort **eTable 5.** Cell-Count-Adjusted Associations of Educational Attainment, Parental Education, and Educational Mobility With Biological Aging in Offspring and Gen3 Framingham Study Participants **eTable 6.** Cell-Count Adjusted Associations of Biological Aging, Educational Attainment, and Educational Mobility With Overall Survival in the Framingham Offspring Cohort **eTable 7.** Smoking-Adjusted Associations of Educational Attainment, Parental Education, and Educational Mobility With Biological Aging in Offspring Framingham Study Participants **eTable 8.** Smoking-Adjusted Associations of Biological Aging, Educational Attainment, and Educational Mobility With Overall Survival in the Framingham Offspring Cohort **eTable 9.** Associations of Educational Mobility, DunedinPACE, and Mortality Using Unstandardized Variables Based on Raw Years of Education **eFigure 1.** Intrafamilial Education Correlation Matrices **eFigure 2.** Associations of Educational Mobility and DunedinPACE by Social Origins in Offspring and Gen3 Framingham Study Participants **eFigure 3.** Association of Educational Mobility With DunedinPACE

Supplement 2. **eTable 4.** Tests of DNA-Methylation Biological Aging Measures as Mediators of Educational Gradients in Mortality Risk

Supplement 3.
Data Sharing Statement
