# Supplementary material for: Educational Mobility, Pace of Aging, and Lifespan Among Participants in the Framingham Heart Study
Source: JAMA Netw Open. 2024 Mar 1;7(3):e240655. doi: 10.1001/jamanetworkopen.2024.0655 (PMC10907927; doi:10.1001/jamanetworkopen.2024.0655)

Supplemental Online Content

Graf GJ, Aiello AE, Caspi A, et al. Educational mobility, pace of aging, and lifespan among participants in the Framingham Heart Study. *JAMA Netw Open.* 2024;7(2):e240655. doi:10.1001/jamanetworkopen.2024.0655

eMethods. Supplemental Methods

eTable 1. Associations of Educational Attainment, Parental Education, and Educational Mobility With Biological Aging in Offspring and Gen3 Framingham Study Participants

eTable 2. Fixed-Effects Regression of Biological Aging on Education Within Sibling Clusters

eTable 3. Associations of Biological Aging, Educational Attainment, and Educational Mobility With Overall Survival in the Framingham Offspring Cohort

eTable 5. Cell-Count-Adjusted Associations of Educational Attainment, Parental Education, and Educational Mobility With Biological Aging in Offspring and Gen3 Framingham Study Participants

eTable 6. Cell-Count Adjusted Associations of Biological Aging, Educational Attainment, and Educational Mobility With Overall Survival in the Framingham Offspring Cohort

eTable 7. Smoking-Adjusted Associations of Educational Attainment, Parental Education, and Educational Mobility With Biological Aging in Offspring Framingham Study Participants

Table 8. Smoking-Adjusted Associations of Biological Aging, Educational Attainment, and Educational Mobility With Overall Survival in the Framingham Offspring Cohort

eTable 9. Associations of Educational Mobility, DunedinPACE, and Mortality Using Unstandardized Variables Based on Raw Years of Education

eFigure 1. Intrafamilial Education Correlation Matrices

eFigure 2. Associations of Educational Mobility and DunedinPACE by Social Origins in Offspring and Gen3 Framingham Study Participants

eFigure 3. Association of Educational Mobility With DunedinPACE

This supplemental material has been provided by the authors to give readers additional information about their work.

© 2024 Graf GJ et al. *JAMA Network Open.*

eMethods. Supplemental Methods

Construction of educational mobility variables

Changes over time in social and policy conditions have contributed to a secular trend of increasing educational attainment across birth cohorts (20). Within each of the three FHS cohorts, participants represent a wide range of birth years. As a consequence, participant's levels of educational attainment reflect both temporal trends and individual differences in achievement. To adjust for the temporal trend and focus measurement on individual achievement, we converted each participant's completed years of education into z-scores standardized to gender and 5-year birth cohort: First, we assigned each participant to a five-year sex-specific birth cohort (e.g. 1901-1905, 1906-1910). Next, we converted reported levels of educational attainment into completed years of education, following the method used by Liu and colleagues (21) and calculated the mean and standard deviation of years of education for each of these groups. Finally, we used these parameters to convert participants' years of education to z-scores.

Mean and standard deviation of educational attainment (in years) are shown in the table below:

Birth Cohort	1885	1890	1895	1900	1905	1910	1915	1920	1925	1930	1935	1940	1945	1950	1955	1960	1965	1970	1975	1980
**Men (n=6258)**																				
n	22	279	342	326	406	420	485	254	302	335	376	437	416	419	452	442	371	238	128	78
mean	11.2	11.4	11.5	11.8	12.1	12.2	12.6	13.2	13.7	14.1	14.6	14.9	15.2	15.2	14.7	14.8	15.1	15.2	15.6	15
SD	2.1	2.2	2.1	2.2	2.3	2.3	2.3	2.6	2.6	2.4	2.4	2.5	2.3	2.1	2	2	1.9	1.8	1.8	1.8
**Women (n=7578)**																				
n	33	342	402	414	478	533	561	245	326	359	376	497	557	491	507	511	426	277	150	93
mean	11.4	11.4	11.6	11.7	12	12.1	12.5	12.9	13.2	13.7	13.9	14.2	14.7	15	15	15	15.4	15.5	15.8	16
SD	1.7	1.9	1.9	2	2.2	2.2	2.1	2.1	2.2	2.2	2.1	2.2	2.5	2.2	1.9	1.8	1.8	1.7	1.6	1.4

© 2024 Graf GJ et al. *JAMA Network Open.*

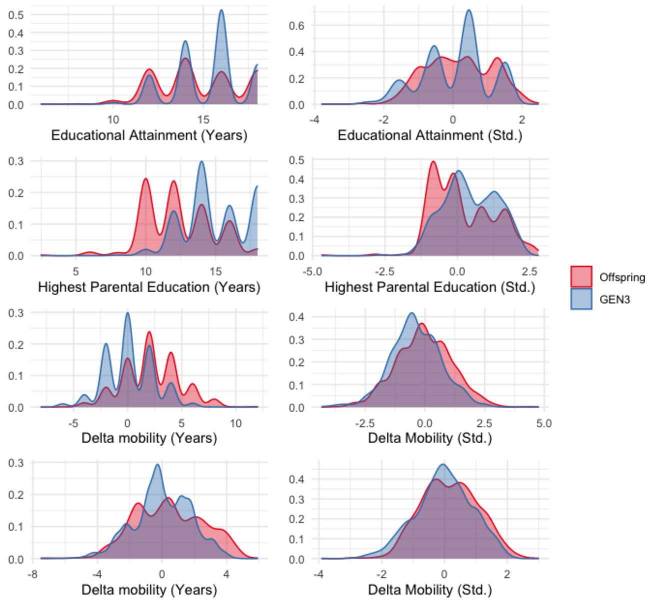

The first two rows of the figure (left) show the distribution of unstandardized and sex- and birth-cohort-standardized educational attainment and parental educational attainment in the Offspring and Gen3 cohorts of the Framingham Heart Study. The last row of the figure shows mobility distribution based on subtracting parents' raw years of education from that of participants (left), as well as subtracting the standardized measures (right). Both Offspring and Gen3 cohort members were upwardly educationally mobile: Offspring cohort members achieved an average of 3 more years of education than their highest-achieving parent, while Gen3 cohort members achieved an average of 0.4 more years of education than their highest-achieving parent.

In the Offspring cohort for whom comprehensive mortality follow-up was available, participants with a faster Pace of Aging were more likely to die (HR 1.61, 95%CI=[1.49,1.74], p<0.001) than those with a slower Pace of Aging. Effect-sizes were similar to those previously reported (Belsky et al. mortality HR=1.65 [1.51-1.79]).

DNA-methylation data

DNA methylation (DNAm) was measured from whole-blood buffycoat samples collected at Visit 8 (2005-2008) for the Offspring Cohort and visit 2 (2009-2011) for the Gen3 cohort using the Infinium HumanMethylation450 BeadChip (Illumina). Processing and normalization of DNAm data has been described previously [2]. Briefly, data were normalized using the "dasen" method in the 'wateRmelon' R package30 and subjected to downstream QC. Samples with missing rate >1% at p<0.01, poor SNP matching to the 65 SNP control probe locations, and outliers by multidimensional scaling techniques were excluded. Probes with missing rate of >20% at p<0.01 were also excluded.

© 2024 Graf GJ et al. *JAMA Network Open.*

DNAm clocks

DNAm clocks are algorithms that combine information from DNAm measurements across the genome to quantify variation in biological age[3].

The first-generation DNAm clocks were developed from machine-learning analyses comparing samples from individuals of different chronological age. These clocks were highly accurate in predicting the chronological age of new samples and also showed some capacity for predicting differences in mortality risk, although effect-sizes tend to be small and inconsistent across studies[4–6]. We analyzed the first-generation clocks proposed by Horvath and Hannum[4,5].

The second-generation DNAm clocks were developed with the goal of improving quantification of biological aging by focusing on differences in mortality risk instead of on differences in chronological age[7,8]. These clocks also include an intermediate step in which DNAm data are fitted to physiological parameters. The second-generation clocks are more predictive of morbidity and mortality as compared with the first-generation clocks[9] and are proposed to have improved potential for testing impacts of interventions to slow aging[10]. We analyzed the second-generation clocks proposed by Lu et al. (GrimAge clock) and Levine et al. (PhenoAge clock)[7,8].

A third generation of DNAm clocks measure pace of aging. In contrast to first- and second-generation DNAm clocks, which aim to quantify how much aging has occurred up to the time of measurement, pace-of-aging clocks aim to quantity how fast the process of aging-related deterioration of system integrity is proceeding[11]. We analyzed the newest pace-of-aging measure, DunedinPACE, which is shorthand for "Pace of Aging Computed from the Epigenome"[12]. DunedinPACE was developed by modeling within-individual multi-system physiological change across four timepoints in the Dunedin Study 1972–1973 birth cohort. Measurements were taken when participants were aged 26, 32, 38 and 45 years. DunedinPACE was developed from analysis of a pace-of-aging composite of slopes of aging-related change measured across this four-timepoint interval in the following measurements: ApoB100/ApoA1 ratio, BMI, blood urea nitrogen, high-sensitivity C-reactive protein, cardiorespiratory fitness, dental caries experience, total cholesterol, forced expiratory volume in 1 second, forced expiratory volume in 1 second/fixed vital capacity ratio, estimated glomerular filtration rate,

© 2024 Graf GJ et al. *JAMA Network Open.*

hemoglobin A1C, high-density lipoprotein cholesterol, leptin, lipoprotein(a), mean arterial pressure, mean periodontal attachment loss, triglycerides, waist-to-hip ratio and white blood cell count. The DunedinPACE DNAm algorithm was derived from elastic net regression of the pace-of-aging composite on Illumina EPIC array DNAm data derived from blood samples collected at the age 45 measurement occasion. The set of CpG sites included in the DNAm dataset used to develop the DunedinPACE algorithm was restricted to those showing acceptable test–retest reliability as determined by Sugden et al.[13].

The key difference between DunedinPACE and the other clocks is that DunedinPACE is designed to measure the pace of aging, a rate of change phenotype, whereas the other clocks were designed to measure biological age, a static level phenotype. To use a car's dashboard as reference, DunedinPACE is designed to function like the speedometer, whereas the other clocks are designed to function as odometers. In some cases, this may lead DunedinPACE to be more sensitive to certain exposures or interventions as compared with other clocks [14,15]. However, in the context of the current study, we anticipate that, at least in the Offspring Cohort, sufficient aging-related changes will have accumulated between the completion of education and the time of DNAm sampling (mean age 66y) that this should not be an issue in our study.

© 2024 Graf GJ et al. *JAMA Network Open.*

eTables

eTable 1. Associations of educational attainment, parental education, and educational mobility with biological aging in Offspring and Gen3 Framingham Study participants. Effect-sizes are age- and sex-adjusted Pearson's r correlations estimated from linear regression, and are interpretable as the standard-deviation unit increase in biological aging or pace or aging associated with a 1-SD increase in educational attainment or educational mobility. Educational attainment and mobility appear to have modest but statistically significant effects on the Pace of Aging in both Offspring and Gen3 cohorts.

	Offspring (n=1652)			Gen3 (n=1449)		
**Predictor**	**ES**	**CI**	**p**	**ES**	**CI**	**p**
**DunedinPACE**						
Educational Attainment	-0.18	(-0.23,-0.14)	<0.001	-0.23	(-0.28,-0.18)	<0.001
Parental Education	-0.07	(-0.12,-0.03)	<0.001	-0.17	(-0.22,-0.11)	<0.001
Mobility (Delta)	-0.06	(-0.10,-0.02)	0.0016	-0.07	(-0.11,-0.02)	0.0061
Mobility (RC)	-0.17	(-0.22,-0.12)	<0.001	-0.21	(-0.26,-0.15)	<0.001
**Horvath Clock**						
Educational Attainment	-0.01	(-0.06,0.04)	0.7861	0.05	(0.00,0.10)	0.0702
Parental Education	-0.04	(-0.09,0.00)	0.0658	0.02	(-0.03,0.07)	0.4767
Mobility (Delta)	0.03	(-0.01,0.07)	0.1445	0.03	(-0.02,0.07)	0.2962
Mobility (RC)	0.01	(-0.04,0.07)	0.6536	0.05	(-0.01,0.10)	0.0888
**Hannum Clock**						
Educational Attainment	-0.01	(-0.06,0.04)	0.7511	-0.02	(-0.07,0.03)	0.5056
Parental Education	-0.02	(-0.06,0.02)	0.3535	-0.01	(-0.06,0.04)	0.6994
Mobility (Delta)	0.01	(-0.03,0.05)	0.5575	-0.01	(-0.05,0.04)	0.7823
Mobility (RC)	0.00	(-0.05,0.05)	0.9726	-0.02	(-0.07,0.04)	0.5601
**PhenoAge Clock**						
Educational Attainment	-0.04	(-0.09,0.01)	0.0811	-0.08	(-0.14,-0.03)	0.002
Parental Education	-0.04	(-0.09,0.00)	0.0516	-0.04	(-0.10,0.01)	0.1426
Mobility (Delta)	0.01	(-0.03,0.05)	0.7368	-0.04	(-0.09,0.01)	0.1195
Mobility (RC)	-0.03	(-0.08,0.02)	0.2814	-0.08	(-0.14,-0.02)	0.005
**GrimAge Clock**						
Educational Attainment	-0.20	(-0.24,-0.15)	<0.001	-0.25	(-0.30,-0.20)	<0.001
Parental Education	-0.08	(-0.12,-0.04)	<0.001	-0.15	(-0.20,-0.10)	<0.001
Mobility (Delta)	-0.07	(-0.11,-0.03)	<0.001	-0.10	(-0.15,-0.05)	<0.001
Mobility (RC)	-0.19	(-0.24,-0.14)	<0.001	-0.24	(-0.29,-0.19)	<0.001

© 2024 Graf GJ et al. *JAMA Network Open.*

eTable 2. Fixed-effects regression of biological aging on education within sibling clusters. Effect-sizes are interpretable as the standard-deviation unit increase in biological-age advancement or pace of aging associated with a 1-SD increase in educational attainment for among siblings who share the same parents. Because siblings who share the same parents share the same expected educational attainment, results provide further evidence that educational mobility is associated with slower biological aging.

Outcome	ES	CI	p
**Overall (N=2437, n=887)**			
DunedinPACE	-0.24	(-0.29,-0.19)	<0.001
Horvath Clock	0.02	(-0.03,0.07)	0.5057
Hannum Clock	-0.04	(-0.09,0.01)	0.1652
PhenoAge Clock	-0.07	(-0.12,-0.02)	0.0099
GrimAge Clock	-0.24	(-0.29,-0.20)	<0.001
**Offspring (N=1096, n=448)**			
DunedinPACE	-0.21	(-0.28,-0.13)	<0.001
Horvath Clock	0.00	(-0.07,0.08)	0.9012
Hannum Clock	0.04	(-0.03,0.12)	0.2671
PhenoAge Clock	0.00	(-0.08,0.08)	0.9301
GrimAge Clock	-0.19	(-0.27,-0.12)	<0.001
**Gen3 (N=1341, n=439)**			
DunedinPACE	-0.25	(-0.32,-0.19)	<0.001
Horvath Clock	0.07	(0.00,0.14)	0.0461
Hannum Clock	-0.02	(-0.08,0.05)	0.5605
PhenoAge Clock	-0.10	(-0.16,-0.03)	0.0045
GrimAge Clock	-0.24	(-0.30,-0.18)	<0.001

© 2024 Graf GJ et al. *JAMA Network Open.*

eTable 3. Associations of biological aging, educational attainment, and educational mobility with overall survival in the Framingham Offspring cohort. The table shows age-adjusted hazard ratios estimated from Cox proportional hazards regression. Hazard ratios are interpretable as the change in mortality risk associated with a 1-SD increase in the pace of aging, in educational attainment, or educational mobility. Slower biological aging, educational attainment, and upward educational mobility are associated with overall survival.

	Offspring Education Sample (n=2411, 607 deaths)			Mediation Sample (n=1648, 402 deaths)		
**Predictor**	**HR**	**CI**	**p**	**HR**	**CI**	**p**
**Biological aging measures**						
DunedinPACE	1.61	(1.49,1.74)	<0.001	1.54	(1.41,1.69)	<0.001
Horvath Clock	1.06	(0.99,1.14)	0.1089	1.10	(1.01,1.20)	0.0284
Hannum Clock	1.24	(1.15,1.34)	<0.001	1.28	(1.17,1.40)	<0.001
PhenoAge Clock	1.32	(1.23,1.43)	<0.001	1.35	(1.23,1.49)	<0.001
GrimAge Clock	1.80	(1.67,1.94)	<0.001	1.76	(1.60,1.92)	<0.001
**Education measures**						
Educational Attainment	0.87	(0.80,0.94)	<0.001	0.87	(0.79,0.95)	0.0028
Parental Education	–	–	–	0.95	(0.86,1.05)	0.3087
Mobility (Delta)	–	–	–	0.92	(0.83,1.02)	0.0996
Mobility (RC)	–	–	–	0.87	(0.79,0.96)	0.0047

© 2024 Graf GJ et al. *JAMA Network Open.*

eTable 4. Tests of DNA-methylation biological aging measures as mediators of educational gradients in mortality risk. The table shows results of mediational analysis following the approach of Valeri and Vanderweele (2013). The first table row shows the controlled direct effect (CDE), second row shows the pure natural direct effect (PNDE), third row shows the total natural direct effect (TNDE), fourth row shows the pure natural indirect effect (PNDE), fifth row shows the total natural indirect effect (TNIE), sixth row shows the total effect (TE), last row shows the proportion mediated (PM). For estimates of the controlled direct effect, the value of the mediator (biological-age) is set to zero.Because there is no established gold standard of biological aging, we repeated our primary analysis of the pace of aging measure using four additional DNA-methylation aging "clocks" which have similarly been shown to predict morbidity and mortality in diverse samples, and which have been shown to be sensitive to a range of socioenvironmental exposures. Note that the total effect is equal to the sum of the pure natural direct effect and the total natural indirect effect (PNDE + TNIE), and to the sum of the total natural direct effect and the pure natural indirect effect (TNDE + PNIE).

See Excel file (Supplement 2).

© 2024 Graf GJ et al. *JAMA Network Open.*

eTable 5. Cell-count-adjusted associations of educational attainment, parental education, and educational mobility with biological aging in Offspring and Gen3 Framingham Study participants. Effect-sizes are age- and sex-adjusted Pearson's r correlations estimated from linear regression, and are interpretable as the standard-deviation unit increase in pace of aging associated with a 1-SD increase in educational attainment or educational mobility. Educational attainment and mobility appear to have modest but statistically significant effects on the Pace of Aging in both Offspring and Gen3 cohorts.

	Offspring (n=1652)			Gen3 (n=1449)		
**Predictor**	**ES**	**CI**	**p**	**ES**	**CI**	**p**
**DunedinPACE**						
Educational Attainment	-0.18	(-0.22,-0.13)	<0.001	-0.20	(-0.25,-0.16)	<0.001
Parental Education	-0.08	(-0.12,-0.04)	<0.001	-0.14	(-0.19,-0.09)	<0.001
Mobility (Delta)	-0.05	(-0.09,-0.01)	0.0066	-0.07	(-0.11,-0.02)	0.0031
Mobility (RC)	-0.16	(-0.21,-0.11)	<0.001	-0.18	(-0.24,-0.13)	<0.001
**Horvath Clock**						
Educational Attainment	0.01	(-0.04,0.05)	0.8215	0.04	(-0.01,0.09)	0.0895
Parental Education	-0.04	(-0.08,0.01)	0.0844	0.01	(-0.04,0.07)	0.6416
Mobility (Delta)	0.04	(0.00,0.08)	0.0773	0.03	(-0.02,0.07)	0.2501
Mobility (RC)	0.02	(-0.03,0.08)	0.3504	0.05	(-0.01,0.10)	0.0964
**Hannum Clock**						
Educational Attainment	0.00	(-0.05,0.04)	0.8855	0.01	(-0.04,0.05)	0.7352
Parental Education	-0.03	(-0.07,0.01)	0.0964	0.01	(-0.04,0.05)	0.7727
Mobility (Delta)	0.03	(-0.01,0.06)	0.1607	0.00	(-0.04,0.04)	0.9521
Mobility (RC)	0.01	(-0.03,0.06)	0.6066	0.01	(-0.04,0.05)	0.7923
**PhenoAge Clock**						
Educational Attainment	-0.04	(-0.08,0.01)	0.1366	-0.07	(-0.12,-0.02)	0.0085
Parental Education	-0.05	(-0.09,-0.01)	0.0221	-0.03	(-0.08,0.02)	0.2799
Mobility (Delta)	0.02	(-0.02,0.06)	0.3873	-0.03	(-0.08,0.01)	0.1413
Mobility (RC)	-0.02	(-0.07,0.03)	0.5065	-0.07	(-0.12,-0.01)	0.0142
**GrimAge Clock**						
Educational Attainment	-0.18	(-0.22,-0.14)	<0.001	-0.23	(-0.27,-0.18)	<0.001
Parental Education	-0.08	(-0.12,-0.04)	<0.001	-0.12	(-0.17,-0.07)	<0.001
Mobility (Delta)	-0.06	(-0.10,-0.02)	0.0015	-0.10	(-0.14,-0.06)	<0.001
Mobility (RC)	-0.17	(-0.22,-0.12)	<0.001	-0.22	(-0.27,-0.17)	<0.001

© 2024 Graf GJ et al. *JAMA Network Open.*

eTable 6. Cell-count adjusted associations of biological aging, educational attainment, and educational mobility with overall survival in the Framingham Offspring cohort. The table shows age-adjusted hazard ratios estimated from Cox proportional hazards regression. Hazard ratios are interpretable as the change in mortality risk associated with a 1-SD increase in the pace of aging, in educational attainment, or educational mobility. Slower biological aging, educational attainment, and upward educational mobility are associated with longer overall survival.

	Offspring Education Sample (n=2411, 607 deaths)			Mediation Sample (n=1648, 402 deaths)		
**Predictor**	**HR**	**CI**	**p**	**HR**	**CI**	**p**
**Biological aging measures**						
DunedinPACE	1.55	(1.42,1.68)	<0.001	1.49	(1.35,1.64)	<0.001
Horvath Clock	1.10	(1.01,1.19)	0.0204	1.15	(1.05,1.26)	0.004
Hannum Clock	1.18	(1.09,1.28)	<0.001	1.23	(1.12,1.36)	<0.001
PhenoAge Clock	1.26	(1.16,1.36)	<0.001	1.28	(1.16,1.42)	<0.001
GrimAge Clock	1.76	(1.62,1.90)	<0.001	1.70	(1.54,1.87)	<0.001
**Education measures**						
Educational Attainment	0.88	(0.81,0.95)	<0.001	0.89	(0.81,0.98)	0.0169
Parental Education	–	–	–	0.95	(0.86,1.05)	0.3169
Mobility (Delta)	–	–	–	0.94	(0.85,1.04)	0.2549
Mobility (RC)	–	–	–	0.90	(0.82,0.99)	0.0298

© 2024 Graf GJ et al. *JAMA Network Open.*

eTable 7. Smoking-adjusted associations of educational attainment, parental education, and educational mobility with biological aging in Offspring Framingham Study participants. Effect-sizes are age- and sex-adjusted Pearson's r correlations estimated from linear regression, and are interpretable as the standard-deviation unit increase in pace of aging associated with a 1-SD increase in educational attainment or educational mobility. Educational attainment and mobility appear to have modest but statistically significant effects on the Pace of Aging.

	Offspring (n=1646)		
**Predictor**	**ES**	**CI**	**p**
**DunedinPACE**			
Educational Attainment	-0.15	(-0.20,-0.11)	<0.001
Parental Education	-0.07	(-0.11,-0.03)	0.0013
Mobility (Delta)	-0.05	(-0.08,-0.01)	0.0175
Mobility (RC)	-0.14	(-0.19,-0.09)	<0.001
**Horvath Clock**			
Educational Attainment	0.00	(-0.05,0.05)	0.8749
Parental Education	-0.04	(-0.09,0.00)	0.0718
Mobility (Delta)	0.03	(-0.01,0.07)	0.1289
Mobility (RC)	0.02	(-0.04,0.07)	0.5766
**Hannum Clock**			
Educational Attainment	0.00	(-0.05,0.05)	0.9753
Parental Education	-0.02	(-0.06,0.02)	0.3706
Mobility (Delta)	0.02	(-0.02,0.06)	0.4272
Mobility (RC)	0.01	(-0.04,0.06)	0.7429
**PhenoAge Clock**			
Educational Attainment	-0.03	(-0.08,0.02)	0.2554
Parental Education	-0.04	(-0.09,0.00)	0.0743
Mobility (Delta)	0.01	(-0.03,0.06)	0.4854
Mobility (RC)	-0.01	(-0.07,0.04)	0.6245
**GrimAge Clock**			
Educational Attainment	-0.13	(-0.17,-0.09)	<0.001
Parental Education	-0.06	(-0.10,-0.02)	0.0012
Mobility (Delta)	-0.04	(-0.07,-0.01)	0.0211
Mobility (RC)	-0.12	(-0.16,-0.08)	<0.001

© 2024 Graf GJ et al. *JAMA Network Open.*

Table 8. Smoking-adjusted associations of biological aging, educational attainment, and educational mobility with overall survival in the Framingham Offspring cohort. The table shows age-adjusted hazard ratios estimated from Cox proportional hazards regression. Hazard ratios are interpretable as the change in mortality risk associated with a 1-SD increase in the pace of aging, in educational attainment, or educational mobility. Slower biological aging, educational attainment, and upward educational mobility are associated with longer overall survival.

	Offspring Education Sample (n=2408, 606 deaths)			Mediation Sample (n=1646, 401 deaths)		
**Predictor**	**HR**	**CI**	**p**	**HR**	**CI**	**p**
**Biological aging measures**						
DunedinPACE	1.57	(1.45,1.70)	<0.001	1.50	(1.36,1.65)	<0.001
Horvath Clock	1.06	(0.99,1.14)	0.104	1.10	(1.01,1.20)	0.0297
Hannum Clock	1.24	(1.15,1.34)	<0.001	1.29	(1.18,1.41)	<0.001
PhenoAge Clock	1.31	(1.22,1.41)	<0.001	1.34	(1.22,1.47)	<0.001
GrimAge Clock	1.84	(1.69,2.00)	<0.001	1.80	(1.62,2.00)	<0.001
**Education measures**						
Educational Attainment	0.88	(0.81,0.95)	<0.001	0.88	(0.80,0.96)	0.0061
Parental Education	–	–	–	0.95	(0.86,1.05)	0.3367
Mobility (Delta)	–	–	–	0.93	(0.84,1.02)	0.1369
Mobility (RC)	–	–	–	0.88	(0.80,0.97)	0.0103

© 2024 Graf GJ et al. *JAMA Network Open.*

eTable 9. Associations of educational mobility, DunedinPACE, and mortality using unstandardized variables based on raw years of education. The table repeats our primary analysis of education, biological aging, and mortality using variables based on raw years of education and unstandardized by sex or birth cohort. The first column shows Pearson's r correlations with the sex- and birth-cohort-standardized version of each measure used in primary analysis. Age- and sex-adjusted associations with DunedinPACE and mortality are shown in the second and third columns. The last column shows results of mediation analysis without exposure-mediator interactions. All effect-sizes were similar to those reported in the main text; associations of primary education measures with DunedinPACE are reported in **eTable 1**, associations of primary education measures with mortality are reported in **eTable 3**, and results of primary mediation analysis are available in **eTable 4**.

	Offspring Cohort				Gen3 Cohort	
Variable (years)	Correlation with standardized measure	Association with DunedinPACE	Association with mortality (HR)	Mediation fraction	Correlation with standardized measure	Association with DunedinPACE
Own education	0.96	-0.18 (-0.23,-0.14)	0.86 (0.78,0.94)	0.50 (0.25,1.11)	0.99	-0.23 (-0.28,-0.17)
Highest parental education	0.95	-0.06 (-0.11,-0.01)	0.97 (0.87,1.07)	1.65 (-5.17,16.42)	0.94	-0.14 (-0.21,-0.08)
Ed. Mobility (Delta)	0.98	-0.09 (-0.14,-0.04)	0.91 (0.82,1.00)	0.36 (-0.91,1.98)	0.96	-0.05 (-0.10, 0.01)
Ed. Mobility (RC)	0.95	-0.17 (-0.22,-0.12)	0.86 (0.78,0.94)	0.46 (0.24,2.34)	0.98	-0.18 (-0.23,-0.12)

© 2024 Graf GJ et al. *JAMA Network Open.*

eFigures

eFigure 1. Intrafamilial education correlation matrices. Histograms on the diagonal line show the distribution of participants' own educational attainment and that of their mother, father, and siblings. Pearson's r correlations are shown above the diagonal line, with corresponding scatterplots and lines of best fit below. Participants' own educational attainment was moderately correlated with their parents' educational attainment (r 0.31-0.36) and highly correlated with their siblings' educational attainment (r 0.73-0.75).

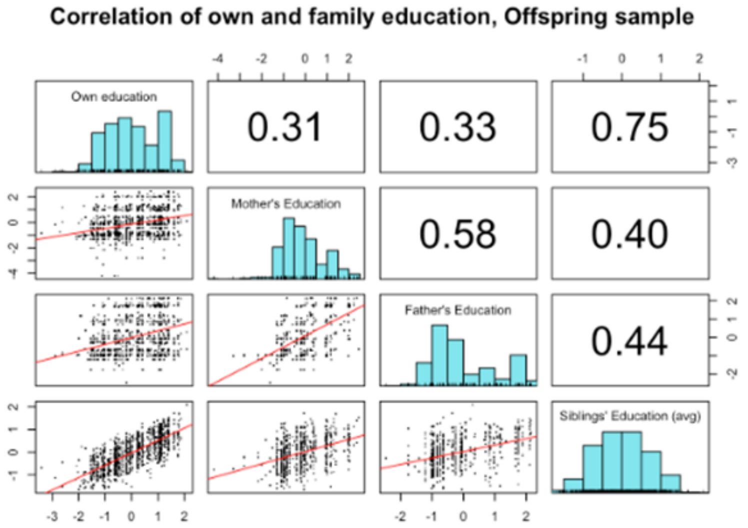

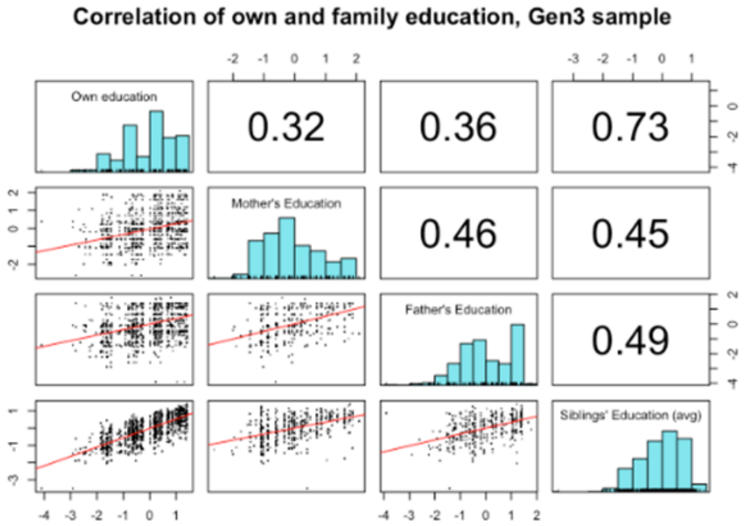

© 2024 Graf GJ et al. *JAMA Network Open.*

eFigure 2. Associations of educational mobility and DunedinPACE by social origins in Offspring and Gen3 Framingham study participants. This figure shows associations of educational mobility with faster pace of aging, stratified by parental educational attainment. Effect-sizes are Pearson's r correlations, denominated in standard-deviation units of pace of aging. Observed returns of educational mobility to healthy aging were observed regardless of social origins, with similar effect-sizes across Offspring and Gen3 Framingham study participants.

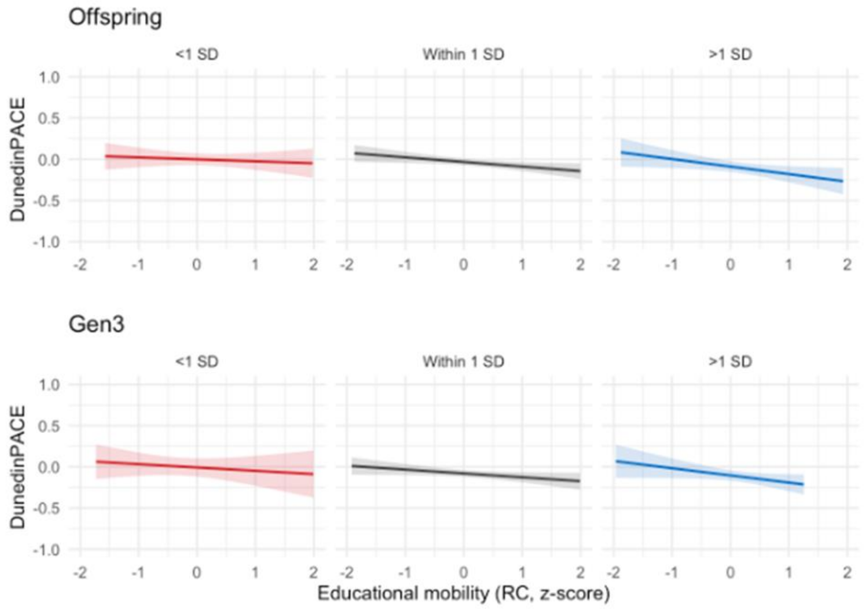

© 2024 Graf GJ et al. *JAMA Network Open.*

eFigure 3. Association of educational mobility with DunedinPACE. The figure shows associations of educational mobility with faster pace of aging. The upper panel shows scatterplots and fitted regression slopes of DunedinPACE on educational mobility, where educational mobility was quantified as the residual of a participant's sex- and birth-cohort-standardized educational attainment z-score on the sex- and birth-cohort-standardized educational attainment z-score of their highest-educated parent (Offspring ES=-0.18, p<0.001; Gen3 ES=-0.22, p<0.001). The lower panel shows scatterplots and fitted regression slopes of DunedinPACE on educational mobility, where educational mobility was estimated using fixed-effect regression of DunedinPACE on sibling differences in educational outcomes (Offspring ES=-0.22, p<0.001; Gen3 ES=-0.25, p<0.001).

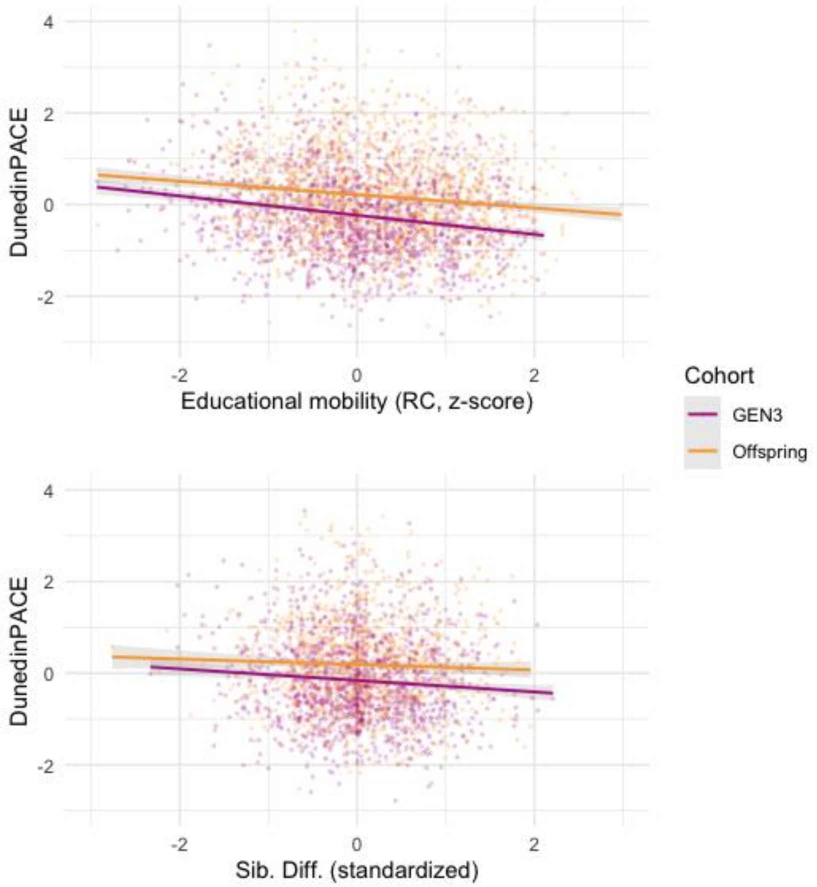

Supplement: Supplement 1. — eMethods. Supplemental Methods eTable 1. Associations of Educational Attainment, Parental Education, and Educational Mobility With Biological Aging in Offspring and Gen3 Framingham Study Participants eTable 2. Fixed-Effects Regression of Biological Aging on Education Within Sibling Clusters eTable 3. Associations of Biological Aging, Educational Attainment, and Educational Mobility With Overall Survival in the Framingham Offspring Cohort eTable 5. Cell-Count-Adjusted Associations of Educational Attainment, Parental Education, and Educational Mobility With Biological Aging in Offspring and Gen3 Framingham Study Participants eTable 6. Cell-Count Adjusted Associations of Biological Aging, Educational Attainment, and Educational Mobility With Overall Survival in the Framingham Offspring Cohort eTable 7. Smoking-Adjusted Associations of Educational Attainment, Parental Education, and Educational Mobility With Biological Aging in Offspring Framingham Study Participants eTable 8. Smoking-Adjusted Associations of Biological Aging, Educational Attainment, and Educational Mobility With Overall Survival in the Framingham Offspring Cohort eTable 9. Associations of Educational Mobility, DunedinPACE, and Mortality Using Unstandardized Variables Based on Raw Years of Education eFigure 1. Intrafamilial Education Correlation Matrices eFigure 2. Associations of Educational Mobility and DunedinPACE by Social Origins in Offspring and Gen3 Framingham Study Participants eFigure 3. Association of Educational Mobility With DunedinPACE [file jamanetwopen-e240655-s001.pdf]
