# Supplementary material for: Educational Mobility, Pace of Aging, and Lifespan Among Participants in the Framingham Heart Study
Source: JAMA Netw Open. 2024 Mar 1;7(3):e240655. doi: 10.1001/jamanetworkopen.2024.0655 (PMC10907927; doi:10.1001/jamanetworkopen.2024.0655)
Supplement: Supplement 3. — Data Sharing Statement [file jamanetwopen-e240655-s003.pdf]

Data Sharing Statement

Graf. Educational Mobility, Pace of Aging, and Lifespan Among Participants in the Framingham Heart Study. *JAMA Netw Open*. Published February 29, 2024.
doi:10.1001/jamanetworkopen.2024.0655

Data
Data available: No
